# An Attenuated Strain of Human Cytomegalovirus for the Establishment of a Subviral Particle Vaccine

**DOI:** 10.3390/vaccines10081326

**Published:** 2022-08-16

**Authors:** Steffi Krauter, Nicole Büscher, Eric Bräuchle, Samira Ortega Iannazzo, Inessa Penner, Nadine Krämer, Patricia Gogesch, Simone Thomas, Marina Kreutz, Mario Dejung, Anja Freiwald, Falk Butter, Zoe Waibler, Bodo Plachter

**Affiliations:** 1 Institute for Virology, University Medical Center of the Johannes Gutenberg-University Mainz, D-55131 Mainz, Germany; 2 Division of Immunology, Section 3/1 “Product Testing of Immunological Biomedicines”, Paul-Ehrlich-Institut, D-63225 Langen, Germany; 3 Leibniz Institute for Immunotherapy, Regensburg and Klinik und Poliklinik für Innere Medizin III, Hämatologie und Internistische Onkologie, University Hospital Regensburg, D-93053 Regensburg, Germany; 4 Proteomics Core Facility, Institute of Molecular Biology, D-55128 Mainz, Germany

**Keywords:** human cytomegalovirus, vaccine, subviral particles, dense bodies, conditional expression, ddFKBP, IE1/IE2, UL25

## Abstract

Human cytomegalovirus (HCMV) infection is associated with severe disease conditions either following congenital transmission of the virus or viral reactivation in immunosuppressed individuals. Consequently, the establishment of a protective vaccine is of high medical need. Several candidates have been tested in preclinical and clinical studies, yet no vaccine has been licensed. Subviral dense bodies (DB) are a promising vaccine candidate. We have recently provided a GMP-compliant protocol for the production of DB, based on a genetically modified version of the HCMV laboratory strain Towne, expressing the pentameric complex of envelope protein gH-gL-pUL128-131 (Towne-UL130rep). In this work, we genetically attenuated Towne-UL130rep by abrogating the expression of the tegument protein pUL25 and by fusing the destabilizing domain ddFKBP to the N-terminus of the IE1- and IE2-proteins of HCMV. The resulting strain, termed TR-VAC, produced high amounts of DB under IE1/IE2 repressive conditions and concomitant supplementation of the viral terminase inhibitor letermovir to the producer cell culture. TR-VAC DB retained the capacity to induce neutralizing antibodies. A complex pattern of host protein induction was observed by mass spectrometry following exposure of primary human monocytes with TR-VAC DB. Human monocyte-derived dendritic cells (DC) moderately increased the expression of activation markers and MHC molecules upon stimulation with TR-VAC DB. In a co-culture with autologous T cells, the TR-VAC DB-stimulated DC induced a robust HCMV-specific T cell-activation and –proliferation. Exposure of donor-derived monocytic cells to DB led to the activation of a rapid innate immune response. This comprehensive data set thus shows that TR-VAC is an optimal attenuated seed virus strain for the production of a DB vaccine to be tested in clinical studies.

## 1. Introduction

The human cytomegalovirus (HCMV), a member of the *β-herpesviridae,* is a pathogen that may cause severe clinical manifestations in patients with immature or compromised immune defense functions. Congenital HCMV infection (cCMV) is frequently associated with perinatal disease conditions and lasting sequelae [1,2,3]. The development of a vaccine against cCMV has thus been defined as a top-priority medical goal [4,5]. The reactivation of HCMV from latency, in addition, is a severe complication of both solid organ and hematopoietic stem cell transplantation [6,7]. The establishment of a vaccine for the prevention of HCMV-related complications in these settings is also highly desirable [8].

Numerous vaccine candidates have been established and tested in preclinical or clinical studies (reviewed in [9,10]). There is still no licensed vaccine available, and the appropriate formulations are debated (reviewed in [9,10,11,12,13,14,15,16,17]). There appears to be consensus, however, that the tegument protein pp65 should be included as a major T lymphocyte antigen. The envelope glycoproteins gB (gpUL55) and gH (gpUL75) have been identified as prominent targets of neutralizing antibodies (nt-abs) and are thus candidate antigens for an HCMV vaccine [18,19,20]. More recently, evidence was provided that the pentameric protein complex (PC) of HCMV envelope proteins, consisting of gH, gL, and pUL128-131, is a major target of the nt-abs response against HCMV, thus supporting the concept of PC inclusion as a component of a future HCMV vaccine [21,22,23].

We and others provided evidence that a vaccine based on subviral particles of HCMV, known as dense bodies (DB), is highly immunogenic [23,24,25,26,27,28,29,30,31,32,33]. DB are synthesized in infected fibroblast cell cultures and are released from these cells at late stages of HCMV replication, concomitant with the release of virions [34,35]. DB are devoid of viral capsids and are therefore non-infectious [36]. Unique features of DB are: (i) that they contain relevant antigens of the T-lymphocyte as well as the nt-abs response, (ii) that they enter cells via fusion of their viral envelope similar to infectious virions, (iii) that they induce both activation and maturation of immature dendritic cells, (iv) and that they can be purified in large amounts from infected cells [16,23]. DB synthesis is particularly efficient following infection of fibroblasts with HCMV laboratory strains. These laboratory strains, however, lack the expression of the PC. In previous work, we restored the PC expression in the HCMV laboratory strain Towne by genetically repairing the mutated UL130 open reading frame (orf, Towne-UL130rep) [23]. More recent work focused on the establishment of a safe and effective way of producing a DB vaccine in a producer cell line, based on the strain Towne-UL130rep. As one safety feature, the UL25 orf was deleted, resulting in Towne-UL130rep-ΔUL25. The pUL25 is an abundant tegument protein that can surprisingly be deleted without hampering DB synthesis [24]. As it is conserved between HCMV strains, it is likely an important protein for viral infection in vivo. In addition, Towne-UL130rep-ΔUL25 proved to be more sensitive to IFN-β treatment. Thus, abrogation of pUL25 expression leads to marked attenuation of HCMV. Furthermore, we have established that the addition of the viral terminase inhibitor, letermovir (LMV), decouples virion from DB synthesis in infected cells, thus allowing DB production while drastically reducing the release of infectious virus from cells [16]. Finally, a protocol for large-scale GMP-compliant production of DBs was established that included inactivation of residual contaminating virus by UV-irradiation [16].

The work presented here focused on providing a final seed virus strain for DB vaccine production. For this, we modified the UL25 orf in Towne-UL130rep-ΔGFP by replacing the wt-sequence with a version in which we deleted all ATG-start codons and replaced most with stop codons. In addition, we modified the virus such that the important IE1/IE2 genes of HCMV were conditionally expressed by fusion with the destabilizing domain ddFKBP [37]. The final seed virus TR-VAC was characterized with respect to its attenuation in vitro, and TR-VAC DB were evaluated with respect to their impact on hematopoietic cells and their immunogenicity in comparison to the known responses induced by Towne-UL130rep [16,23].

## 2. Materials and Methods

### 2.1. Cells, BAC-Cloning, and Viruses

Human foreskin fibroblasts (HFF) were cultured as described before [38]. All HCMV variants used in this analysis were derived from bacterial artificial chromosome (BAC) clones. For downstream cloning, the recently established parental strain Towne-UL130repΔGFP was used [23]. The cloning procedures were performed based on the bacterial galactokinase (*galK*) positive/negative selection as described by Warming et al. [39]. The *galK* gene, which initially was inserted for the depletion of GFP in the BAC cassette, was seamlessly deleted as described in [16].

For the generation of Towne-UL130repΔGFP-UL25ms, the *galK* gene was first removed from the GFP locus, using flanking oligonucleotides and *galK* negative selection [39]. In a second step, the *galK* gene was re-inserted, thereby deleting the UL25 coding sequence. In a third step, the UL25 multiple-stop (ms) sequence, purchased from Synbio Technologies, Monmouth Junction, USA was re-inserted, thereby removing the *galK* gene (Figure S1).

For the final cloning steps to generate TR-VAC, the *galK* gene was inserted directly 5′-to the ATG start codon of UL123/122 (IE1/IE2). The resulting BAC-clones were then finally modified by replacing the *galK* gene with ddFKBP, generating a fused open reading frame encoding ddFKBP-IE1/IE2. The ddFKBP peptide is an unstable variant of the FK506- and rapamycin-binding protein (FKBP). Fusion of the ddFKBP peptide to a heterologous protein allows the conditional regulation of the levels of that protein by adding or omitting the stabilizing ligand Shield-1 to cell cultures [40].

Virus reconstitution from BAC-clones was achieved by transfecting column purified BAC-DNA (Plasmid Purification Kit; Machery&Nagel, Düren, Germany) into HFF with Superfect transfection reagent (Qiagen, Hilden, Germany) as previously described [24]. Viral stocks were generated by passaging transfected HFF until all cells showed a typical cytopathic effect. For the production of TR-VAC stocks, Shield-1 was added in a final concentration of 2 µM to the culture medium. The supernatants were then collected and used as seed stocks. Supernatants were frozen at −80 °C until further use. Viral stocks of TR-VAC were generated in the presence of Shield-1 (1–2 µM, supplemented every 48 h, Aobious, Köln, Germany).

### 2.2. Production and Purification of Virions and DB

DB of HCMV were prepared as previously described [24]. HFF were infected with culture supernatants containing the virus of interest. For the LMV experiments, 50 nM of the substance were added to the cell culture at the time of infection and 3 days after initial infection. For cultures with Shield-1, the substance was added at 2 µM.

Culture supernatants from infected HFF were collected 1 week after infection and, after removal of cellular debris, pelleted via ultracentrifugation. After resuspension, the different components of the resulting pellet were fractionated via glycerol-tartrate density gradient ultracentrifugation [35]. Subsequently, DB were collected, concentrated via centrifugation, and stored at −80 °C until further use. For the production of TR-VAC DB, HFF were infected with TR-VAC and were cultured in the absence of Shield-1. Supernatants of the cells were harvested 1 week after initial infection and processed as described above. LMV was added in some of the experiments.

### 2.3. SDS-PAGE, Silver Staining/Instant Blue, and Immunoblotting

The protein composition of purified DB was analyzed by Sodium Dodecyl Sulfate-Polyacrylamide Gel Electrophoresis (SDS-PAGE), followed by either silver staining with silver nitrate or by immunoblotting, respectively. For silver staining, 2 µg of virions or DB per lane were loaded on a 10% Tris-Glycine-polyacrylamide gel. The gels were subsequently fixed and stained using the Roti^®^-Black P silver staining kit for proteins (Carl Roth, Karlsruhe, Germany). For immunoblotting, lysates from 1 × 10^5^ cells per lane were loaded on a 10% SDS-Bis-Tris-polyacrylamide gel. Following electrophoresis, the proteins were transferred to a Polyvinylidene Difluoride (PVDF) membrane (Immobilon-FL, Millipore, Billerica, MA, USA). The antibody p63-27, provided by William Britt, was used for IE1-staining [41,42]. For loading control, an antibody directed against Glycerinaldehyd-3-phosphat-Dehydrogenase (GAPDH; Sigma via Merck, Darmstadt, Germany) was used. For visualization, a rabbit anti-mouse-antibody conjugated with horse-radish-peroxidase (HRP, Dako, Santa Clara, CA, USA) was used.

### 2.4. Analysis of Infectious Virus by IE1-Staining

The determination of the levels of infectious virus in culture supernatants, collected at 8 days post infection (dpi), or within DB preparations was performed by staining with monoclonal antibody p63-27. For this, HFFs were seeded on 96-well flat-bottom culture plates (BD Falcon, Heidelberg, Germany) at a density of 5 × 10^3^ cells/well in minimal essential medium (MEM) containing 5% fetal calf serum (FCS) for 24 h prior to the addition of virus or DB in the presence of 2 µM Shield-1. 48 h after infection, cells were washed in PBS and fixed with 96% ethanol for 20 min at room temperature. After a further washing step, cells were incubated for 1 h at 37 °C with 50 µL hybridoma supernatant of monoclonal antibody p63-27. Binding of the IE1-specific antibody was detected with a horse-radish peroxidase (HRP)-coupled polyclonal rabbit anti-mouse secondary antibody (Dako, Santa Clara, CA, USA). Antibody binding was visualized by incubation with a 3-amino-9-ethylcarbazole (AEC) solution. After another washing step, the numbers of IE1-positive nuclei were counted in the microscope. The mean of IE1-positive cells counted in six (undiluted samples) to eight (diluted samples) was taken as the relative measure of infectivity.

### 2.5. Quantitative Real-Time PCR Analysis

To quantify viral genomes accumulating within infected cells or released into the supernatant during infection, a quantitative real-time PCR using the TaqMan technology was performed. HFF were infected with infectious culture supernatants that were normalized for an uptake of 4 viral genomes per cell. 2 µM Shield-1 was added every 48 h in some samples. At each of the following 9 days, viral genomes were isolated from 10^5^ infected cells or from 200 µL aliquots of the supernatant, using the high pure viral nucleic acid kit (Roche Diagnostics, Rotkreuz, Swizerland) according to the manufacturer’s recommendations. TaqMan PCR was performed in 50 µL reaction mixtures, containing 5 µL of either the viral DNA sample or standard DNA solution. Additional components were 2 units HotStarTaq DNA polymerase (Qiagen, Hilden, Gemany), 15 pmol of each primer, and 5 pmol probe directed against the HCMV gene UL54, which was labelled with 6-carboxy-fluorescein reporter dye and 6-carboxy-tetramethyl-rhodamine quencher dye. The DNA standard for quantification was prepared by serial dilutions of 10^5^ to 10 copies of cosmid pCM1049 [43]. For thermal cycling, one initial step of 1 min at 95 °C was followed by 42 amplification cycles consisting of 15 s at 95 °C and 1 min at 60 °C. Real-time PCR was performed using an ABI Prism 7700 sequence detector (PE Applied Biosystems, Weiterstadt, Germany). For each DNA sample, PCR analysis was carried out in triplicates.

### 2.6. Immunization of Rabbits and Neutralization Assays

Immunizations of rabbits were performed by Eurogentec according to all Federation for Laboratory Animal Science Associations (FELASA) recommendations (https://secure.eurogentec.com/product/research-animal-care.html (accessed on 20 June 2022)). For the quantitation of nt-abs levels, neutralization assays were performed. In brief, serum samples were put on the bench at RT for 30 min. Twofold serial dilutions of the serum were incubated with an equal volume of virus and incubated for 4 h at 37 °C in a 5% CO_2_ atmosphere. To determine remaining infectivity, the virus-serum mixture was added to HFF, which were seeded with a density of 1.5 × 10^4^ cells/well on gelatin-pretreated (0.1%) 96-well flat-bottom culture plates (BD Falcon, Heidelberg, Germany). Upon 24 h of incubation at 37 °C and 5% CO_2_, the supernatants were removed and the cells stained for IE1 expression, using the antibody p63-27 and a HRP-coupled secondary antibody (Dako, via Agilent, Santa Clara, CA, USA). The number of positive cells was counted in quadruple replicates. The mean value of the four replicates was divided by the mean value calculated from eight replicates of the positive control (no serum added) and defined as residual infectivity. The residual infectivity value was determined for each serum dilution. The 50% neutralization titer (NT50) was defined as the serum dilution that resulted in an infectivity of 50% or less (i.e., neutralization of 50% or more of infectious virus).

### 2.7. Human Monocyte and T Cell Isolation from Buffy Coats

The isolation and differentiation of immune cells was performed as previously described [44]. In brief, human buffy coats from healthy donors were obtained by the German Red Cross blood donation center (Frankfurt am Main, Germany). For the analyses of HCMV-specific T cell responses, buffy coats with defined HCMV-serostatus were provided. Buffy coats were used for the isolation of peripheral blood mononuclear cells (PBMCs) by Pancoll (PAN-Biotech GmbH, Aidenbach, Germany) density gradient centrifugation. Monocytes were directly isolated from PBMCs by positive selection using CD14 MicroBeads (Miltenyi Biotech, Bergisch Gladbach, Germany) according to the manufacturer’s instructions. Autologous T cells were isolated from thawed PBMCs (stored for six days at −80 °C in FCS plus 10% DMSO) by negative selection using a Pan T cell isolation kit (Miltenyi Biotech) according to the manufacturer´s instructions.

#### Human Monocyte Isolation and DB Treatment

Monocytes were cultured in 6-well plates. 1 × 10^6^ monocytes were incubated in 1 mL RPMI without human sera for 30 min. Afterwards, RPMI was removed and the cells were exposed to 20 μg/100 μL PBS of UV-inactivated DB. As a control, monocytes were incubated with PBS. After 2 h, 880 μL RPMI+ 20 μL AB serum were added to the cells. Following 24 h incubation, the supernatant was collected, centrifuged, and stored at −20 °C for further use. Monocytes were scraped off and washed in 500 mL PBS. After centrifugation, PBS was removed and the cell pellets were stored at −80 °C until they were processed for mass spectrometry analyses.

### 2.8. Differentiation and Stimulation of Human Monocyte-Derived Dendritic Cells

Isolated monocytes (1 × 10^6^ cells/mL) were cultured in X-VIVO 15 serum-free medium (with L-glutamine, gentamicin and phenol red; Lonza, Basel, Switzerland) in the presence of 1000 U/mL granulocyte-macrophage colony-stimulating factor (GM-CSF) and 1000 U/mL interleukin (IL)-4 (both CellGenix, Freiburg, Germany) for five days for dendritic cell (DC)-differentiation. In vitro differentiated DC were stimulated with 0.1 mg/mL lipopolysaccharid (LPS; *Salmonella abortus equi*, Sigma-Aldrich, St. Louis, MO, USA) plus 1000 U/mL IFN-γ (PeproTech Inc., Rocky Hill, NJ, USA), 1 µg/mL TR-VAC DB, or 1 µg/mL Towne-UL130rep DB (TR DB). Untreated cells served as controls. Upon 24 h of incubation, cells were harvested with cold Ca^2+^- and Mg^2+^-free PBS containing 1 mM EDTA followed by flowcytometric analyses.

### 2.9. Co-Culture of DC and T Cells

Co-cultures of DC and autologous T cells were performed as previously described (Miller et al. 2018, PMID: 29554701). Briefly, differentiated DC were harvested with cold Ca^2+^- and Mg^2+^-free PBS containing 1 mM EDTA and seeded in 100 µL X-VIVO 15 per 96-well round-bottom plate (1.25 × 10^4^ DC/well). DC were stimulated with 200 µg/mL CMV peptide pool (Mabtech AB, Nacka Strand, Sweden), 1 µg/mL TR-VAC DB, or 1 µg/mL TR DB. Untreated cells served as controls. 24 h after stimulation, autologous T cells (1 × 10^6^ T cells/mL), stained with 4 µM carboxyfluorescein succinimidyl ester (CFSE; Invitrogen, Carlsbad, CA, USA), were added in 100 µL to the pre-treated DC. The co-culture was incubated for six additional days at 37 °C followed by harvesting of the T cells and flowcytometric analyses.

### 2.10. Flow Cytometry

For flowcytometric analysis, harvested cells were stained for 20 min at 4 °C using the following antibodies: anti-human CD3-Allophycocyanin (APC) (clone UCHT1, BD Pharmingen, San Jose, CA, USA), anti-human CD4- Phycoerythrin (PE) (clone RPA-T4, BD Pharmingen), anti-human CD8-PacificBlue (clone RPA-T8, BD Pharmingen), anti-human CD14-PacificBlue (clone M5E2, BD Pharmingen), anti-human CD40-PacificBlue (clone 5C3, BioLegend, Fell, Germany), anti-human CD69-APC-Cyanine (Cy)7 (clone FN50, BioLegend), anti-human CD80-APC (clone MEM-233, EuroBioSciences, Friesoythe, Germany), anti-human CD83- Fluorescein (FITC) (clone HB15e, BD Pharmingen), anti-human CD86-PE (clone IT2.2, BioLegend), anti-human HLA-ABC-PacificBlue (clone W6/32, BioLegend), and anti-human HLA-DR-APCCy7 (clone L243, BioLegend). Flowcytometric analyses were performed using a LSRFortessa flow cytometer. Data were analyzed using the BD FACSDiva software version 8.0.3 (BD Biosciences, Heidelberg, Germany) or FlowJo software version 10.7.1 (Tree Star, Ashland, Wilmington, DE, USA).

### 2.11. Mass Spectrometry and Data Analyses

#### 2.11.1. Sample Preparation

For mass spectrometry analyses 1 × 10^6^ monocytes were lysed in 50 μL 2x Laemmli buffer without bromophenol blue staining and heated at 99 °C for 10 min. The samples were stored at −20 °C until they were passed to the proteomics core facility (AG Butter, Proteomics Core Facility, IMB Mainz) for MS measurement. Therefore, the samples were thawed and mixed with 1x NuPAGE LDS Sample Buffer (Life technologies, Carlsbad, CA, USA) and 100 mM DTT. Finally, the samples were incubated at 70 °C for 10 min.

The proteins were run on the 10% SDS PAGE for 10 min at 180 V to allow the proteins to move into the resolving gel; then, proteins were fixated in the gel for 15 min in a 7% acetic acid/40% methanol solution and subsequently stained for 15 min with a solution of 0.25% Coomassie Blue G-250 (Biozym, Hessisch Oldendorf, Germany), 7% acetic acid, and 45% ethanol. The SDS PAGE gels were washed several times with deionized water on the shaker to remove excess dye. The in-gel digestion was performed in principle as previously described [45] and detailed as follows. Each gel lane was cut separately using the new sharp scalpel and then minced and transferred to an Eppendorf tube. Gel pieces were de-stained (50% ethanol in 25 mM NH_4_HCO_3_) for 15 min to remove the Coomassie dye. The supernatant was removed, and the gel pieces were dehydrated by adding 100% acetonitrile for 10 min on the rotator. The acetonitrile was dispensed off by pipetting and the samples were dried to completion using a vacuum evaporator (Eppendorf). The dried samples were rehydrated and disulfide bonds in the proteins were reduced using a reduction buffer (10 mM DTT in 50 mM NH_4_HCO_3_ pH 8.0) for 1 h at 56 °C. The buffer was removed by pipetting and cysteine residues of proteins were subsequently alkylated with 50 mM iodoacetamide in 50 mM NH_4_HCO_3_ pH 8.0 for 45 min at room temperature in the dark. Samples were dehydrated again by adding 100% acetonitrile and dried by vacuum evaporation. The vacuum-dried gel slices were incubated with 1 μg trypsin per tube in 50 mM triethylammonium bicarbonate buffer pH 8.0 at 37 °C overnight. Digested peptides were extracted twice by adding 150 μL of 30% acetonitrile, and the supernatant containing the digested peptides was transferred to the new Eppendorf tube. For the second time, the peptide extraction was done by adding 150 μL of 100% acetonitrile to the gel pieces for 15 min at 25 °C agitating at 1400 rpm in a Thermo shaker (Eppendorf) and the supernatant was transferred to the previous Eppendorf tube. The reductive demethylation step was performed as described [46]. Each sample pair including a replicate were switched with the dimethyl labels. Equal amounts of peptides from all labelled samples were mixed and the purification and desalting of the peptides was done using the C18 stage-tips (M3 company as previously described) [47]. The eluted peptides were loaded on the silica column of 75 μm inner diameter (New Objective, FS360-75-8-N-5-C30) packed to 25 cm length with 1.9 μm C18 Reprosil beads (Dr. Maisch GmbH, Ammerbuch, Germany) using the EasyLC1000 liquid chromatography (Thermo, Waltham, WA, USA).

Peptides were separated on the C18 column using an EasyLC1000 HPLC (Thermo) with the following 4 h reversed-phase chromatography gradient: 0–4 min, 2–5% solvent B; 4–157 min, 5–22% solvent B; 157–208 min, 22–40% solvent B; 208–212 min, 40–95% solvent B; 212–217 min, 95% solvent B; 217–221 min, 95–2% solvent B; and 221–225 min, 2% solvent B (solvent A: 0.1% formic acid, solvent B: 80% acetonitrile containing 0.1% formic acid) and directly sprayed into a Q-Exactive Plus mass spectrometer (Thermo Scientific, Waltham, WA, USA) for the data acquision. The mass spectrometer was operated in the positive ion scan mode with a full scan resolution of 70,000; AGC target 3 × 106; max IT = 20 ms; and scan range 300–1650 *m*/*z* with a top10 MS/MS DDA method. Normalized collision energy was set to 25 and MS/MS scan mode operated at a resolution of 17,000; AGC target 1 × 105, and max IT of 120 ms.

#### 2.11.2. MaxQuant Settings

For database searching, MaxQuant (V 1.5.2.8 and V 1.6.10.43 [48]) was used with default settings. Trypsin/P was set as digestion mode allowing for 2 missed cleavages. In terms of further settings, variable modifications included Acetyl (Protein N-term) and Oxidation (M); fixed modifications were Carbamidomethyl (C), and FDR of 1% on peptide and protein level was applied. Re-quantify was activated, quantification was based on razor and unique peptides. For labelling, we used a dimethyl label coupled to the lysine residues and N-terminal ends of each peptide. Depending on the experimental design of the experiment, we used a double or triple labelling procedure, where always a standard dimethyl group was used to label the light sample. A modified dimethyl with a mass shift of 4 Dalton was used to label the heavy sample in double labelling and as a medium label in triple label experiments. The heavy label in the triple labelling condition had a mass shift of 8 Dalton.

#### 2.11.3. Database

The search was performed against the protein databases in fasta format from the Uniprot website. The databases were updated once a year and we combined all available sequences from *Homo sapiens*, human cytomegalovirus, as well as the Towne strain from human cytomegalovirus.

#### 2.11.4. Filtering MaxQuant Results

The results produced by MaxQuant were further processed to remove known contaminants (as provided within MaxQuant), reverse database binders (decoy), and protein groups only identified by site modifications. A second filtering step is removing all protein groups with less than 2 razor peptides (1 unique). Due to high similarity between our bait protein and other tegument proteins, the algorithm from MaxQuant accounts the razor peptides to the protein group with the most matching peptide IDs. In this case, we included our bait manually, although the overall ratio for the bait protein might not be accurate, due to missing razor peptide quantification and only based on a single unique peptide. The quantification ratios of the dimethyl labels were log2 transformed and plotted forward against reverse experiment (label switch).

## 3. Results

### 3.1. Cloning of an HCMV Seed Strain for DB-Production

#### 3.1.1. Cloning of TR-VAC

**Figure 1:**
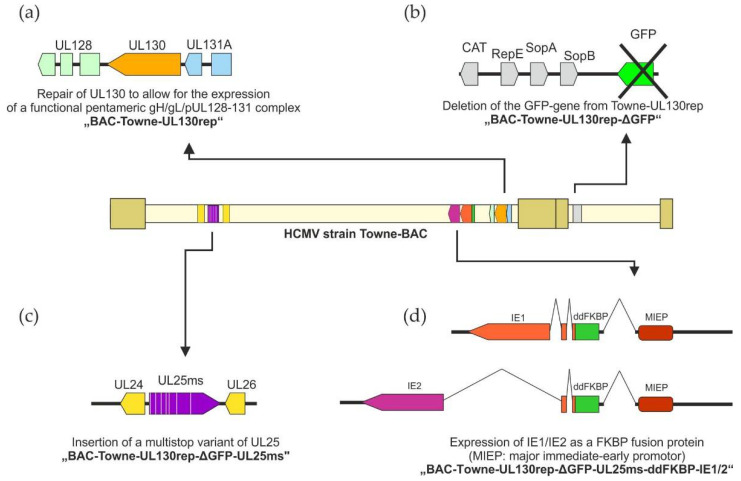
Schematic representation of the cloning strategy of TR-VAC with HCMV Towne-BAC as parental strain [49,50]. All cloning steps were performed using the GalK positive/negative selection system. (**a**) Replacement of the mutated UL130 gene of strain Towne by the respective functional sequence of strain TB40/e resulted in the generation of BAC-Towne-UL130rep [23]. (**b**) Removal of the GFP-coding resulted in the generation of BAC-Towne-UL130rep-ΔGFP [16]. (**c**) Replacement of the UL25 coding sequence by a multi-stop variant of the gene resulting in the generation of BAC-Towne-UL130rep-ΔGFP-UL25ms (see Figure S1). (**d**) Insertion of the ddFKBP destabilizing domain in frame with the UL123/122 open reading frames, encoding HCMV IE1 and IE2 resulting in BAC- Towne-UL130rep-ΔGFP-UL25ms-ddFKBP-IE1/2. Transfection of the latter BAC on HFF resulted in the establishment of strain TR-VAC.

Based on the bacterial artificial clone Towne-BAC [49,50], we recently restored the expression of the PC by replacing the mutated UL130 reading frame in the HCMV Towne strain by the complete pUL130 coding sequence from the HCMV TB40/e strain, thereby generating Towne-UL130rep (Figure 1) [23]. In the next step, we deleted the sequence encoding the green fluorescent protein (GFP) by BAC-recombineering as described before, resulting in Towne-UL130rep-ΔGFP [29]. Based on Towne-UL130rep-ΔGFP, we here replaced the pUL25 coding sequence in Towne-UL130rep-ΔGFP by a variant in which all ATG start codons were mutated (Figure 1 and Figure S1a). The resulting mutant, Towne-UL130rep-ΔGFP-UL25ms (multiple-stop), is disabled in pUL25 expression (Figure S1b).

To establish a final seed strain for DB-vaccine production, we genetically modified the backbone of Towne-UL130rep-ΔGFP-UL25ms, resulting in a seed strain (TR-VAC) that conditionally expresses the IE1/IE2 genes (IL123/UL122). For this, we used a strategy originally introduced by Glass et al. [51], genetically fusing the unstable variant of the FKBP12 protein, termed ddFKBP [52], to the N-terminus of the co-terminal IE1- and IE2-proteins of HCMV by BAC recombineering (Figure 1d). IE1 and IE2 are metabolically destabilized by the ddFKBP fusion during infection with this strain. Growth of infected cells in the presence of the ddFKBP-ligand Shield-1 stabilizes IE1 and IE2, while omission of Shield-1 leads to the depletion of the essential functions of the two proteins [51,52]. The correct insertion of the ddFKBP fragment was verified by nucleotide sequencing (Figure S2). The strain TR-VAC was reconstituted in HFF following transfection of the respective BAC-construct in the presence of Shield-1.

#### 3.1.2. Protein Expression and Replication of TR-VAC

**Figure 2:**
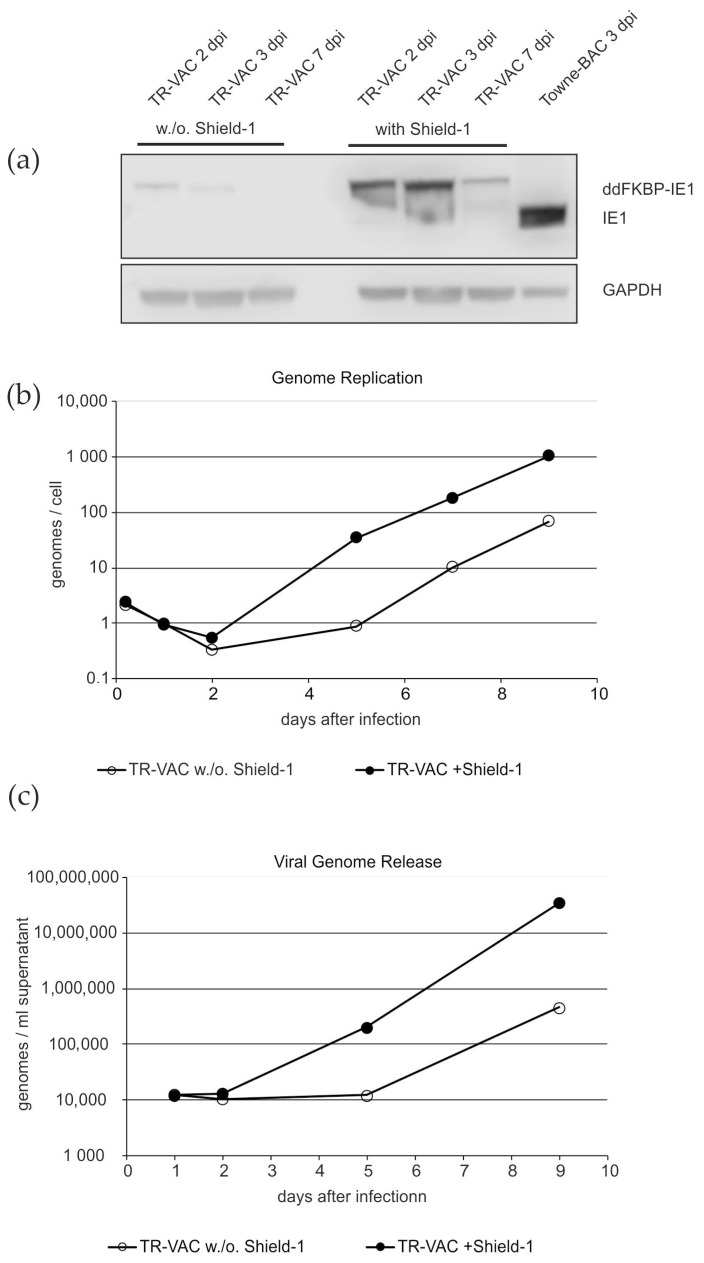
Analysis of ddFKBP-IE1 protein expression and genome replication in, and genome release from TR-VAC infected HFF. (**a**) Immunoblot analysis of lysates of HFF, infected for the indicated incubation periods with TR-VAC or with the parental strain Towne-BAC (control). Shield-1 was either added or omitted. The levels of GAPDH were measured from the same samples as loading controls. (**b**) Quantitative PCR analysis of the viral genome replication in HFF, infected with TR-VAC and cultured with or without Shield-1. Lysates for analysis were collected at the indicated dpi. (**c**) Quantitative PCR analysis of the viral genome release from HFF, infected with TR-VAC and cultured with or without Shield-1. Cell culture supernatants for analysis were collected at the indicated dpi.

Following reconstitution, the IE1-expression levels in TR-VAC infected cells in the presence and absence of Shield-1 was analyzed by Western blot (Figure 2a). For this, HFF were infected with either TR-VAC or Towne-BAC (control). Cells were collected at 2 dpi, 3 dpi, and 7 dpi. Analyses of the cell lysates revealed that very little of the ddFKBP-IE1 protein was expressed without Shield-1-treatment. In contrast, high levels of the fusion protein were detectable at 2 dpi and 3 dpi, with declining levels at 7 days post infection (dpi), in accordance with the known IE1-expression kinetics during HCMV infection. These data showed that the fusion protein of ddFKBP-IE1 was expressed in TR-VAC infected fibroblasts and that the steady-state levels of the protein was sensitive to the addition of Shield-1.

To further characterize TR-VAC sensitivity to Shield-1, viral genome replication was analyzed by quantitative PCR (q-PCR; Figure 2b). HFF were infected with a low multiplicity of infection (m.o.i) of TR-VAC either with or without Shield-1. Cells were collected at different dpi, lysed, and subjected to q-PCR. The results showed that genome replication was impeded by 1 to 1.5 orders of magnitude in the absence of Shield-1, compared with cultures with the substance added. This reduction was in agreement with previous literature showing that IE1-deleted mutants of HCMV show severely impaired replication kinetics [53].

To investigate whether the inhibited replication of TR-VAC in the absence of Shield-1 correlated with reduced viral release, q-PCR was performed with supernatants of infected HFF-cultures (Figure 2c). The release of viral genomic DNA as a surrogate of viral release was reduced by two orders of magnitude, following infection of HFF with TR-VAC in the absence of Shield-1. This corresponded to the results obtained by Glass et al., using a similar ddFKBP-IE1 construct [51].

#### 3.1.3. Confirmation of IFN-β Sensitivity

**Figure 3:**
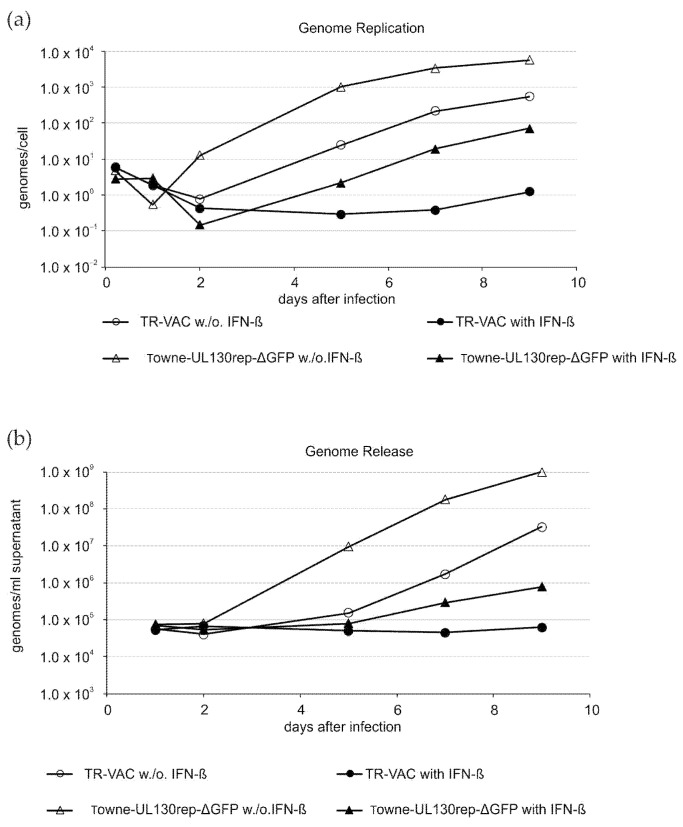
Sensitivity of genome replication and genome release of TR-VAC to IFN-ß treatment. (**a**) Quantitative PCR analysis of the viral genome replication in HFF, infected with TR-VAC or Towne-UL130rep-ΔGFP and cultured with or without IFN-ß. Lysates for analysis were collected at the indicated dpi. (**b**) Quantitative PCR analysis of the viral genome release from HFF, infected with TR-VAC or Towne-UL130rep-ΔGFP and cultured with or without Shield-1. Cell culture supernatants for analysis were collected at the indicated dpi.

The replication of HCMV is sensitive to IFN-ß. To confirm that this sensitivity was retained in TR-VAC, HFF were infected in the presence and the absence of IFN-ß. The strain Towne-UL130rep-ΔGFP was used as a control. Both viral DNA replication and viral genome release in cell cultures infected with TR-VAC proved to be severely reduced by IFN-ß treatment (Figure 3). Interestingly, TR-VAC appeared to be even more sensitive to IFN-ß compared with Towne-UL130rep-ΔGFP. This may be related to the abrogation of pUL25 expression, as deletion of that gene resulted in enhanced sensitivity of the resulting HCMV strain to IFN-ß [24].

Together these results showed that the establishment of TR-VAC provides a seed virus for DB-vaccine production that expresses the PC while being severely attenuated by the abrogation of pUL25 expression and the conditional expression of IE1/IE2 variants.

### 3.2. Induction of nt-abs by TR-VAC DBs in Rabbits

**Figure 4:**
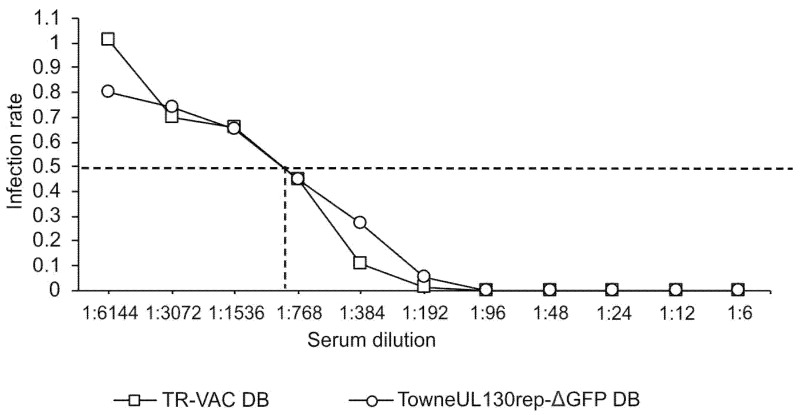
Comparative analysis of the levels of HCMV nt-abs induced in rabbits by intramuscular injection of either TR-VAC DB or TowneUL130rep-ΔGFP DB. Individual sera were serially diluted and tested for neutralization of HCMV strain TR-VAC on HFF indicator cells. The relative levels of neutralization of HCMV were measured by counting nuclei stained positive for IE1 expression. The serum dilution corresponding to 50% neutralization is displayed by dashed lines.

We and others have shown that DBs induce robust nt-abs responses in mice and rabbits [23,31,32,33]. To confirm that the immunogenicity with respect to the humoral immune response was retained in TR-VAC DBs, 2 rabbits were immunized with these particles. DBs of Towne-UL130rep-ΔGFP were used in parallel in two additional animals as a control. The rabbits received 4 intramuscular injections of 100 µg each on days 1, 10, and 18. The blood was collected at day 28 and the serum was used for standard neutralization assays on HFF. The representative result from the sera of one animal each is shown in Figure 4. A 50% neutralization of HCMV was seen at a serum dilution of roughly 1:900 for both DB-preparations, confirming the high potential of DBs from TR-VAC for the induction nt-abs responses.

### 3.3. Analyzing the Immunogenicity of TR-VAC DBs on Human Immune Cells

**Figure 5:**
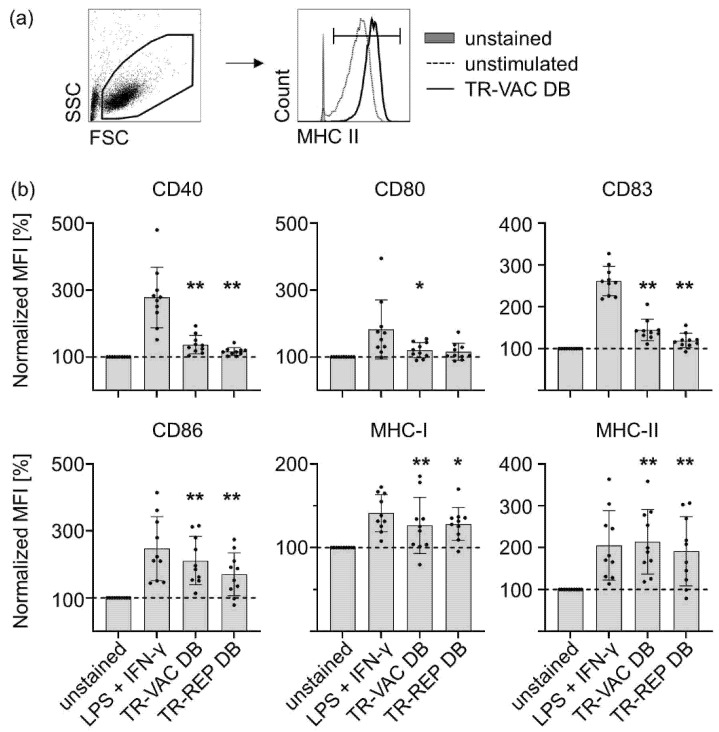
Stimulation of human DC with TR-VAC. Human monocyte-derived DCs were stimulated with TR-VAC DB or TowneUL130rep-ΔGFP DB (TR-REP DB; 10 µg/5 × 10^5^ DC), 1 µg/mL LPS plus 100 U/mL IFN-γ as positive control, or left untreated (Ø) for 24 h. Subsequently, cells were harvested and stained with α-CD40-PacificBlue, α-CD80-APC, α-CD83-FITC, α-CD86-PE, α-MHC-I (HLA-ABC), and α-MHC-II (HLA-DR)-APC-Cy7 antibodies for flow cytometric analyses. (**a**) Representative FACS-analyses of MHC II-expression for one donor is given comparing unstained (gray-shaded histogram), unstimulated (dashed line), and TR-VAC DB-stimulated (black line) DC (peaks normalized to mode). (**b**) Statistical analyses of marker-expression as indicated and described (n = 10). Expression-levels were normalized to the MFI of untreated cells (dashed line). Statistical analysis was performed using Wilcoxon’s matched-pairs signed-rank test. *, *p* < 0.05; **, *p* < 0.01; mean value plus SD.

We additionally analyzed the immunogenicity of the TR-VAC-derived DB with the help of human primary immune cells derived from buffy coats. DC are major antigen presenting cells (APC) and are critical in connecting innate and adaptive immunity. To determine their activation status upon 24 h of stimulation, we analyzed the upregulation of activation and maturation markers on the DC population by flow cytometry, as exemplarily shown for MHC II-expression given in Figure 5a. Treatment of cells with LPS and IFN-γ served as a positive control. Upon 24 h of stimulation with TR-VAC DB, a moderately but significantly enhanced expression could be observed for CD40, CD80, and CD83 (Figure 5b). The expression of CD86, MHC I, and MHC II was significantly enhanced to comparable ranges as observed for stimulation with the positive control. Comparable expression patterns of these markers were detected upon DC stimulation with TR DB. Hence, attenuation of DB did not impair the overall immunogenicity on the level of DC activation. Altogether, these results suggest that TR-VAC DBs induce a moderate activation of in vitro differentiated monocyte-derived human DC.

**Figure 6:**
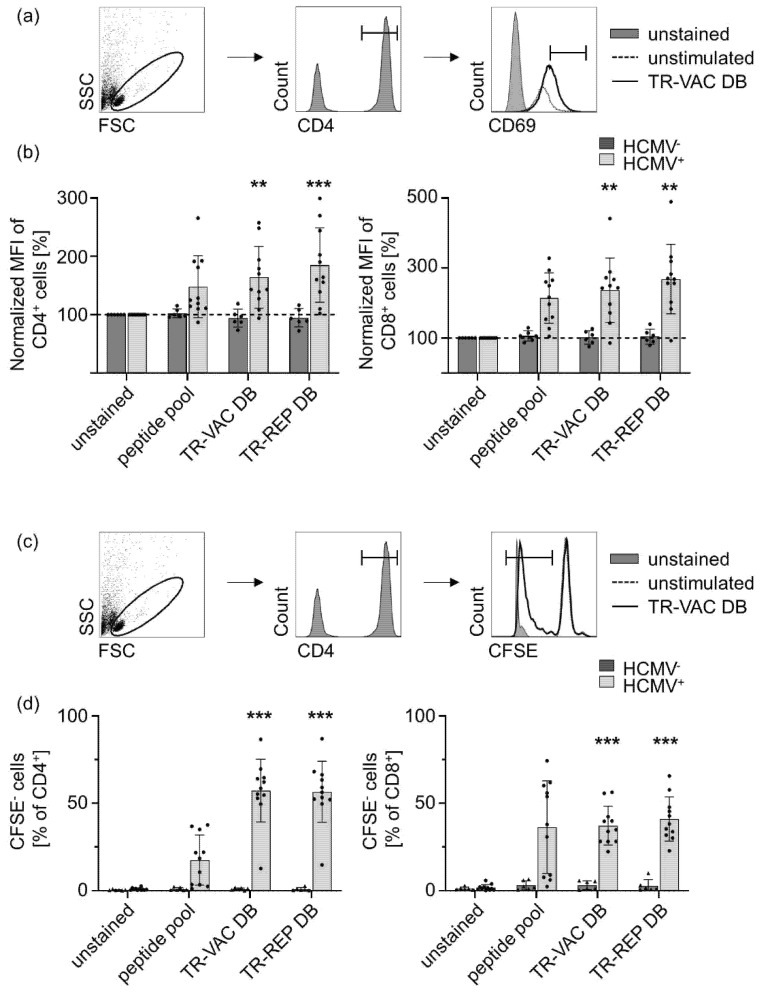
Co-cultivation of TR-VAC-stimulated human DC with HCMV-specific T cells. DC derived from HCMV- and HCMV+ donors were stimulated with TR-VAC DB or TowneUL130rep-ΔGFP (TR) DB (10 µg/5 × 10^5^ DC), 0.4 µg/mL of a commercially available peptide pool as positive control, or were left untreated (Ø) for 24 h. Subsequently, CFSE-labelled autologous PAN T cells were added to treated DCs. After further incubation for 6 days, cells were harvested and stained with α-CD4-PE, α-CD8-PacificBlue, and α-CD69-APC-Cy7 antibodies for flow cytometric analyses. (**a**) Representative FACS-analyses of CD69 expression on CD4+ T cells for one HCMV+ donor is given comparing unstained (gray-shaded histogram), unstimulated (dashed line), and TR-VAC DB-stimulated (black line) cultures (peaks normalized to mode). (**b**) Statistical analyses of CD69 expression on CD4+ (left graph) and CD8+ (right graph) T cells of HCMV- (dark gray bars, n = 6) and HCMV+ (light gray bars, n = 11) donors. (**c**) Representative FACS-analyses of CFSE-dilution within CD4+ T cells for one HCMV+ donor is given comparing unstained (gray-shaded histogram), unstimulated (dashed line), and TR-VAC DB-stimulated (black line) cultures (peaks normalized to mode). (**d**) Statistical analysis of CFSE-dilution within CD4+ (left graph) and CD8+ (right graph) T cells of HCMV- (dark gray bars, n = 6) and HCMV+ (light gray bars, n = 11) donors. Statistical analysis was performed using Wilcoxon’s matched-pairs signed-rank test. **, *p* < 0.01 ***, *p* < 0.001; mean value plus SD.

In the next step, we analyzed whether HCMV-specific adaptive immune responses are induced by DB-stimulated DC. For this, we incubated monocyte-derived human DC with TR-VAC DB and TR-REP (Towne-UL130rep-ΔGFP) DB for 24 h and subsequently co-cultured them with autologous PAN T cells. Upon 6 additional days of incubation, we analyzed the expression of CD69 as a measure of activation of HCMV^-^ and HCMV^+^ CD4^+^ and CD8^+^ T cells (Figure 6a). As a positive control, we used DC stimulated with a peptide pool consisting of 42 defined HCMV-specific immunodominant peptides known to be present on human MHC I and MHC II. Both CD4^+^ and CD8^+^ T cells significantly enhanced CD69 expression upon 5 d of co-culture with the DB-stimulated DC. Importantly, this effect was antigen-specific, as it was observed for T-cells from HCMV^+^-seropositive but not for T-cells from HCMV seronegative donors (Figure 6b). In addition to CD69 expression, we confirmed the activation of HCMV-specific CD4^+^ and CD8^+^ T cells by measuring their proliferation. For this, DB-stimulated DC were co-cultivated with autologous PAN T cells, which were previously stained with CFSE. Upon 6 d of co-cultivation, the dilution of CFSE was determined as a measure of CD4^+^ and CD8^+^ T cell proliferation (Figure 6c). In line with the data obtained for CD69 expression, both T cell subtypes from HCMV^+^ donors strongly proliferated upon co-culture with the DB-stimulated DC. No significant differences in immunogenicity could be observed comparing TR-VAC DB and TR-REP DB, which confirms that the attenuation resulting in TR-VAC did not affect the immunogenic capacities of the derived DB. In contrast, CD4^+^ and CD8^+^ T cells of HCMV^-^ donors hardly proliferated upon co-culture with the DB-treated DC (Figure 6d). Taken together, these data clearly indicate that DC stimulated with TR-VAC- or TR-derived DB are able to induce HCMV-specific T cell activation and proliferation in vitro.

### 3.4. Impact of DB on the Proteome of Primary Monocytes

**Figure 7:**
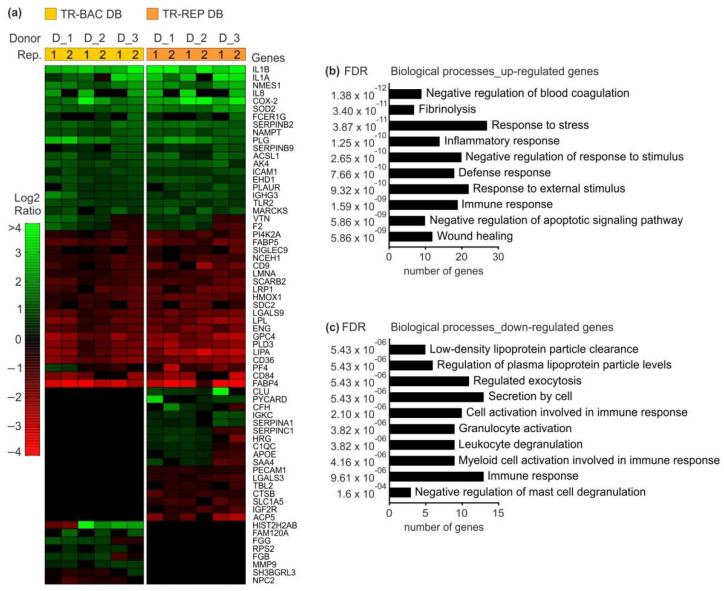
Proteome analysis from monocytes exposed to DB. (**a**) Heatmap of proteins which are differentially regulated in monocytes exposed to both, PC-negative Towne-BAC (TR-BAC DB) and PC-positive TowneUL130rep-ΔGFP (TR-REP) DB. Each row represents a protein and two columns represent the two technical replicates (1;2) of one donor (D_1). Regulated proteins with a minimum Log2Ratio of ±0.58 in at least one technical replicate in every donor were hierarchically arranged. The log2Ratio is represented with a color gradient. Green indicates up-regulation, red indicates down-regulation. Black areas represent values that did not reach the threshold of ±0.58. (B + C) Gene ontology (GO) enrichment analysis, performed using the STRING database (Version 11.5), indicated up- (**b**) and down-regulated (**c**) terms of biological processes. The terms were arranged according to the False Discovery Rate (FDR). The *y*-axis represents biological processes categories with the corresponding *p*-values, while the *x*-axis indicates the number of genes involved in each category. Rep., technical replicate; STRING, Search Tool for the Retrieval of Interacting Genes/Proteins.

To obtain an understanding of cellular changes in monocytes following DB exposure, proteomic analyses were performed. Blood-derived monocytes were incubated with 20 µg of UV-inactivated PC-negative Towne-BAC or PC-positive TowneUL130rep-ΔGFP DB. Following 24 h incubation, monocytes were pelleted and prepared for mass spectrometry analyses. For compilation of the data, analyses from three independent donors were used. The heatmap in Figure 7a shows a hierarchical cluster of 66 proteins that were identified to be regulated in monocytes 24 h after they have been exposed to DB. Proteins that exhibited a Log2Ratio of at least ±0.58 (corresponding to a 1.5-fold change) in at least one of the technical replicates in each donor were considered as differentially regulated compared with PBS-treated monocytes. The color intensity indicates the degree of protein up (green)- or down- (red) regulation. Black areas represent values that did not reach the threshold of ±0.58. Among the 66 regulated proteins, 84.8% are known to be IFN responsive according to the INTERFEROME database [54] of IFN Regulated Genes. To understand the major biological functions of these differentially regulated proteins, we performed gene ontology (GO)- analysis using the STRING (Search Tool for the Retrieval of Interacting Genes) database [55]. The ten most significant up- and down-regulated GO classifications of biological processes are depicted in the bar-charts in Figure 7b,c. The terms and pathways were selected by the smallest false discovery rate. Interestingly, the most distinctly up-regulated proteins, such as interleukin 1 (IL-1), IL-8, or PTGS2, related to GO terms of stress and immune responses. Most of the down-regulated proteins related to GO terms of lipid metabolism and immune response. We also observed that both Towne-BAC and TowneUL130rep-ΔGFP DB stimulated the secretion of the pro-inflammatory mediators IL-6 and TNF-α, but this was inconsistently observed depending on the DB preparation. Thus, the relevance of this finding deserves further investigation (data not shown). Taken together, the findings highlight the properties of DB to activate a rapid innate immune response and therefore boost their potential as a vaccine.

### 3.5. Establishment of a Protocol for DB-Production

**Figure 8:**
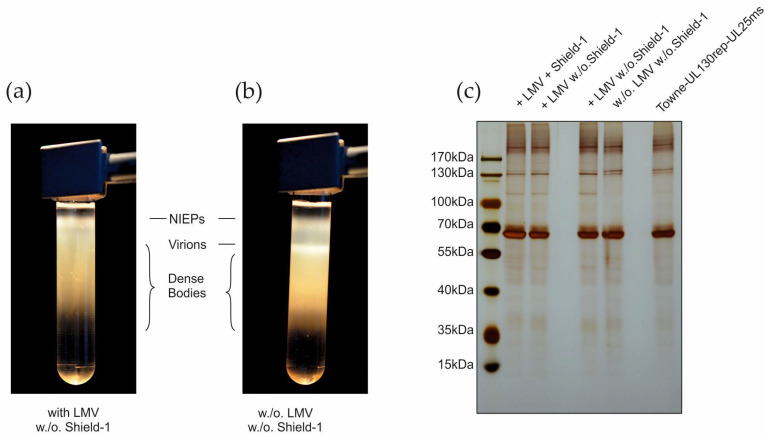
TR-VAC DB production with HFF. (**a**,**b**) Ultracentrifugation gradients of producer cell supernatants showing the fractions of non-infectious enveloped particles (NIEPs), virions, and DB that were that were released from HFF, infected with TR-VAC and grown without Shield-1 and either with (**a**) or without (**b**) LMV. The fractions corresponding to the different fractions are indicated. (**c**) SDS-polyacrylamide gel and silver nitrate staining for the analysis of the DB-fractions from (**a**,**b**). In addition, TR-VAC DB purified from HFF and cultured with both LMV and Shield-1 (left lane) and Towne-UL130rep-UL25ms DB cultured without addition of LMV or Shield-1 (right lane) were analyzed as control. The protein bands were visualized by staining with silver nitrate.

The addition of Shield-1 to TR-VAC infected fibroblast cultures allowed the generation of high-titer virus stocks for further analysis. This protocol is also applicable for the establishment of seed-virus stocks for downstream HCMV production. It was still unclear, however, if TR-VAC infection resulted in amounts of DB suitable for upscaling to a large-scale DB-vaccine production process. To investigate this, different culture conditions were applied in order to establish an appropriate protocol providing the production of an optimized DB-yield. We showed before that LMV addition to HCMV infected fibroblasts almost completely abrogated viral progeny production without impairing high-level DB-release [16]. Here we infected HFF with TR-VAC in the presence or absence of Shield-1 and with or without the viral terminase inhibitor LMV. The cell culture supernatants were collected and used for DB preparation by gradient ultracentrifugation as described [16,35]. Upon ultracentrifugation, the gradients of these supernatants displayed large amounts of DBs (smear) but were devoid of the virion band as visible in the control without LMV and Shield-1 treatment (Figure 8a,b). The band representing non-infectious enveloped particles (NIEPs) was still detectable. Cultures grown with Shield-1 and LMV produced amounts of DB (Table. 1) that were comparable with the amounts obtained after purifying DB from the control virus Towne-UL130rep-UL25ms (not shown). Surprisingly, TR-VAC-infected HFF that were cultured in the absence of Shield-1 produced amounts of DB in a range that will be suitable for upscaling. The yield of TR-VAC DB produced under different conditions and harvested on two consecutive days is shown in Table 1. The DB material was subsequently subjected to SDS-PAGE and stained with silver nitrate (Figure 8c) showing the expected protein pattern of the DB fractions.

**Figure 9:**
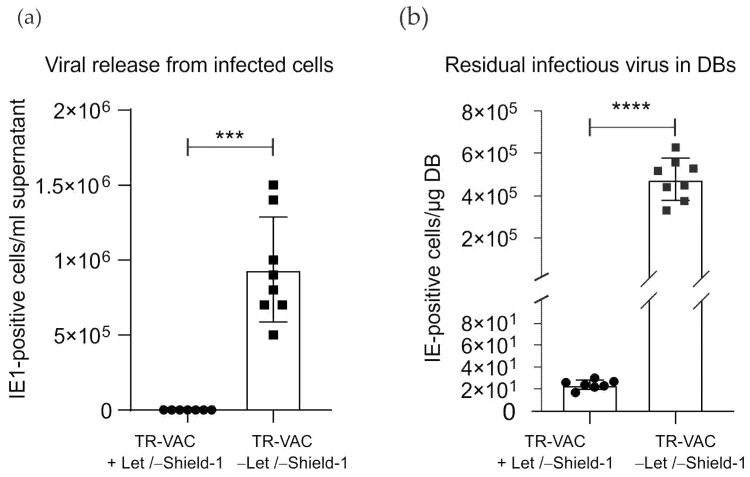
Release of infectious virus from TR-VAC-infected HFF and residual infectious virus in DB preparations. (**a**) Release of infectious virus from TR-VAC-infected cells in the presence or absence of LMV. (**b**) Levels of residual infectious virus in TR-VAC DB, prepared from cultures with or without LMV. The levels of infectious virus in the culture medium (**a**) or DB-preparations (**b**) were measured by serial dilution of the material on HFF indicator cells combined with staining for IE1 and counting of positive nuclei. The assay was performed in the presence of Shield-1 to avoid IE1-degradation (*p* value in a: 0.0001, ***; *p* value in b: <0.0001, ****).

To analyze the residual release of infectious virus from TR-VAC cultures under +LMV/-Shield-1 conditions, the supernatants of such cultures were analyzed using IE1-staining as previously described [42]. No IE1-positive cells were detected when supernatants from +LMV/-Shield-1 TR-VAC cultures were applied to indicator cells and stained with an IE1-specific antibody (Figure 9). In contrast, infectious virus was detectable in -LMV/-Shield-1 cultures.

To investigate the level of viral load in the purified material, DB fractions, isolated upon ultracentrifugation of the producer cell supernatants, were also tested for the induction of IE1-expression in indicator cells (Figure 9b). In this instance, the assay was performed in the presence of Shield-1 to avoid IE1 degradation. Almost no infectious virus was detectable per µg DB, compared with controls, showing the efficiency of the protocol to reduce infectivity in the DB preparation.

**Figure 10:**
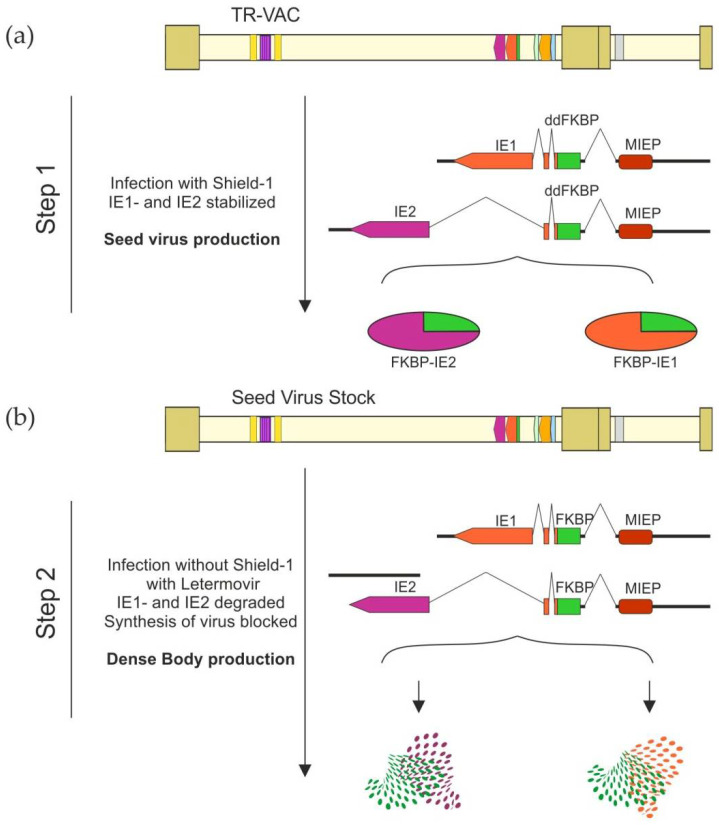
Schematic representation of the production strategy of the TR-VAC DB as a potential vaccine. (**a**) Production of a seed virus. The structures of the ddFKBP-IE1 and ddFKBP-IE2 fusion genes are shown. The splicing events are depicted by the bent light solid lines. The fusion proteins are schematically displayed. (**b**) Production of the TR-VAC DB-based vaccine candidate. HFF are infected with TR-VAC without Shield-1 but in the presence of LMV. This results in the degradation of IE1 and IE2 and diminished viral replication, while DB production is not impaired.

Taken together, the production of TR-VAC-derived DB in the presence of LMV leads to a marked reduction of virus contamination in purified DB samples. In addition, significant amounts of DB are released from TR-VAC-infected cells even in the absence of Shield-1. A simplified scheme for the production of TR-VAC seed virus and TR-VAC-derived DBs as vaccine candidates is illustrated in Figure 10. High-titered stocks of seed virus for vaccine production can be established by the addition of Sheld-1 to HFF cultures, infected with TR-VAC (Figure 10a). The virus from these stocks will then be applied to production cultures in the presence of LMV, but omitting Shield-1. Thus, the latter substance will not be included in the final production process.

## 4. Discussion

The need for the establishment of a vaccine against the consequences of HCMV infection has been emphasized in numerous publications [4,5,6,7,8,9,10,11,12,14,15,17]. Most authors would agree that a vaccine based on one single antigen might not be suitable to address all the requirements a successful vaccine would have to meet. The approach to use HCMV-derived DB as a vaccine is promising in that it combines proteins that have been identified over the years to be important for both humoral and cellular immune responses [15,16]. A challenge of the DB approach, however, is the requirement to produce these particles in cell cultures that have been infected with the respective HCMV seed virus. Thus, safety in terms of preventing contamination of the DB preparation with residual infectious particles is an important issue. We recently provided a protocol for the production of DB compliant for upscaling to generate material for clinical studies [16]. This included the application of LMV to the producer cells to reduce viral loads in purified DB and to use UV-irradiation in the downstream process to remove residual virus. This strategy proved to be effective in removing infectious virus from the DB material. To meet potential concerns about carry-over of infectious virus into the final vaccine, we decided at this point to include additional safety features to the vaccine candidate by genetically attenuating the seed virus for production.

In a first step, the UL25 gene was targeted. Lack of pUL25 in HCMV renders the virus more susceptible to interferons in vitro [24]. This suggests that the interferon response would be more effective in vivo in limiting viral replication compared with wt HCMV. Thus, with this mutation, the unlikely event of infectious contamination within DB preparations would be attenuated, and elimination by the immune response would be facilitated by the lack of UL25. We chose to replace the wt UL25 gene by a sequence where all the start codons were mutated and most of them converted into stop codons. This strategy ensured that the neighboring genes UL24 and UL26 remained unaffected. 

As an additional modification for attenuation, we originally planned to express the essential protein pUL51 in fusion to the ddFKBP destabilizing domain in the context of the HCMV genome [16]. This strategy, however, was unsuccessful, as we could not reconstitute infectious virus after transfection of the modified HCMV-BAC into HFF. We consequently used another approach by fusing the ddFKBP domain to the N-terminus of IE1 and IE2, giving rise to the final seed virus TR-VAC. Glass and colleagues had shown before that this genetic alteration in the HCMV genome renders the resulting virus severely attenuated [51]. In the presence of Shield-1, however, TR-VAC replicates like wt HCMV and, thus, seed virus stocks can easily be generated for subsequent vaccine production.

A similar approach of fusing ddFKBP to IE1/IE2 and pUL51 has been taken by Wang and colleagues, thereby generating a conditionally replication-defective derivative of the laboratory strain AD169 [56]. This virus, termed V160, was the basis for the establishment of a vaccine and was tested in phase I clinical studies. The vaccine was well tolerated and no viral shedding was detected in the recipients [57]. In contrast to this approach, TR-VAC infectious virus is only used for DB production. Based on the results with V160, it is assumed that even in the event of fortuitous carry-over of TR-VAC, viral replication would be prevented by the lack of both IE1/IE2 and pUL25 expression. Thus, in addition to the use of LMV and UV-irradiation in the process of DB production, the prevention of IE1/IE2 and pUL25 expression provides additional features for an optimized safety profile for TR-VAC-derived DB as potential HCMV-vaccine candidates.

As shown before, LMV, a viral terminase inhibitor drastically reduces virion release from HFF without impairing DB synthesis [16]. Similar results were obtained by Schneider-Ohrum and colleagues who used the terminase inhibitor 2-bromo-5,6-dichloro-1-beta-D-ribofuranosyl benzimidazole riboside (BDCRB) and tangential flow filtration for DB production [32]. To ensure maximized DB generation and minimized risk of carry-over virus particles, we initially followed the concept of adding Shield-1 to the cultures to enable early events of HCMV infection and combined this with LMV to block virion synthesis at later stages. Surprisingly, the removal of Shield-1 at later stages of infection did not significantly impair DB yield. Thus, a combination of adding LMV, and removing Shield-1 at late stages of infection, in a production process based on TR-VAC provides a rationale to reduce the viral load in purified DB close to undetectable levels. Considering the fact that a downstream process for production including UV-irradiation already proved to be highly effective in inactivating infectious virus when using wt HCMV, the cultivation conditions with LMV without Shield-1 and using the optimized seed-strain TR-VAC provide various additional features for DB vaccine production on different levels.

The immunogenicity of DB has been demonstrated before [23,24,25,26,27,28,29,30,31,32,33]. With respect to the nt-abs, even better responses were seen when using Towne-UL130rep DB, containing the PC for immunization [23]. Since several genetic modifications had been applied to generate TR-VAC, it was important to confirm the immunogenicity of DB of that strain prior to proceeding to clinical studies. Immunization experiments with TR-VAC DB in rabbits indeed showed that the induction of nt-abs was comparable to that seen after Towne-UL130rep DB immunization. Results comparable to those using DB of other HCMV strains were also found when looking at the cellular immune response. TR-VAC-derived DB induced a moderate activation of monocyte-derived DC. These DB-treated DC were also suitable to induce antigen-specific adaptive immune responses as investigated by co-culture experiments with DB-treated DC and autologous T cells. To be able to depict HCMV-specific T cell responses, we used buffy coats from human donors with a defined HCMV+ serostatus possessing an HCMV-specific immunologic memory. Indeed, the DB-treated DC were able to induce significant HCMV-specific responses of both CD4+ and CD8+ T cells. The very minor proliferation of HCMV+ T cell might suggest the presence of a few antigen-specific T cells according to their physiologic abundance. On this in vitro level, the data confirm the presentation of immunodominant HCMV-specific antigens delivered by the DB by DC. Furthermore, DB induce sufficient activation and maturation of DC to mediate a robust and specific T cell response. Importantly, these analyses indicate that attenuating TowneUL130-rep to generate the seed-strain TR-VAC did not affect the immunogenicity of the respective DB.

Results from mass spectrometry analyses revealed an upregulation of TRL2. TRL2 triggers IFN-I production in human monocytes, via the signaling intermediates TBK1 and IRF3, that induces the expression of a subset of ISGs [58,59]. Indeed, DB stimulation of monocytes altered the abundance of 66 proteins of which 56 are ISGs. Interestingly, and of relevance in the context of a vaccine, DB induced SERPINB9 (Peptidase Inhibitor 9). In vivo, it is known to specifically inhibit Granzyme B (GrB)- mediated cytotoxicity which is associated with subclinical rejection after organ transplantations [60,61,62]. SERPINB9 plays a key role in immunity against CMV infection; its upregulation supports the potential of a DB-based vaccine.

Strikingly, we found a number of down-regulated proteins of the lipid metabolism in monocytes exposed to DB. Previous studies have shown that in HFFs, HCMV increases fatty acid biosynthesis to accumulate lipids that are required for the formation of the envelope, thus are essential for production of infectious virions. Inhibition of the relevant fatty acid biosynthetic enzyme ACC1 (Acetyl-Coenzyme A Carboxylase) blocked the production of progeny virus ([63,64,65]). HCMV does not replicate in monocytes [66,67]. However, monocytes have been identified to harbor latent HCMV which can reactivate upon differentiation of these cells [68,69,70], as reviewed in [71,72]. It is tempting to speculate as to whether DB might impede viral reactivation by interfering with the fatty acid biosynthesis.

Interestingly, two representatives of the fatty acid-binding proteins family (FABPs), namely FABP4 and FABP5, were robustly down-regulated in monocytes incubated with DB. Both proteins are also expressed in macrophages and dendritic cells [73,74]. FABP4 and FABP5 have been shown to coordinate lipid-mediated processes and inflammatory immune response pathways [75]. In macrophages and dendritic cells, FABP4 leads to the activation of the IKK-NF-κB pathway, which is essential to control the inflammatory activity in these cells [76]. FABP4-deficient macrophages display reduced IκB kinase and NF-κB activity, resulting in suppression of inflammatory function [73]. Interestingly, it has been shown that COX-2 synthesis is under NF-κB control. During initial inflammation, NF-κB dimers translocate to the nucleus and induce target genes as COX-2. COX-2 catalyzes the synthesis of pro-inflammatory prostaglandins (PGs) [77,78]. However, during later stages of inflammation, PGs also have anti-inflammatory effects that suppress inflammation by inhibiting NF-κB ([79,80]. Inhibition of NF-κB activity is desirable in case of resolution of inflammation and from an over-stimulation of the immune system. Interestingly, NF-κB is also known to enhance the expression of HCMV immediate-early genes [81,82,83,84]. Thus, inhibition of the IKK-NF-κB pathway by reduced FABP4 and FABP5 levels may both restrict exaggerated inflammatory responses and HCMV replication. Further studies are needed to investigate this in detail.

## 5. Conclusions

In conclusion, DB are a promising vaccine candidate to protect against HCMV infection and disease. A process has been established for the production of DB according to GMP guidelines. With TR-VAC, a seed virus strain has been generated that ideally feeds into this process.

## Figures and Tables

**Table 1:** Yield of TR-VAC DBs following gradient ultracentrifugation.

Letermovir	Shield-1	Purification	Yield [µg]	No. of Flasks [175 cm^2^]
50 nM	2–3 µM	1	491	10
x	x	2	842	10
50 nM	x	1	644	10
50 nM	x	2	465	10

## Supplementary Materials

The following supporting information can be downloaded at: https://www.mdpi.com/article/10.3390/vaccines10081326/s1, Figure S1: Nucleotide sequence and protein patterns of virions and DB of strain Towne-UL130rep-ΔGFP-UL25ms. Figure S2: Nucleotide sequence of the ddFKBP-IE1/2 fusion gene.
