# Supplementary material for: An Attenuated Strain of Human Cytomegalovirus for the Establishment of a Subviral Particle Vaccine
Source: Vaccines (Basel). 2022 Aug 16;10(8):1326. doi: 10.3390/vaccines10081326 (PMC9413975; doi:10.3390/vaccines10081326)

TR-VAC, Figure S1

(a)

```
Towne-UL130r  60580 agcaactgagccgaacgcacctcagcagacgagaggatcgtcgtcgcggcgtcgcagctcgtcacgtcgctctggcgaaccctcgacggtgatttatatcccctcgagcaacgaggacacgccggcggatgaggaggcggaggacagcgttttcacgagcacgcgggcgcgcagcgccacggaagatctggatcgcatggaggccggtttg
UL25ms_Seq        1 ------------------------------------tcgtcgtcgcggcgtcgcagctcgtcacgtcgctctggcgaaccctcgacggtgatttatatcccctcgagcaacgaggacacgccggcggatgaggaggcggaggacagcgttttcacgagcacgcgggcgcgcagcgccacggaagatctggatcgcttggaggccggtttg

Towne-UL130r  60790 tcgccctacagcgtctcctcggacgctccgtcgtccttcgagctcgtgcgcgagaccggcggcaccggcgccgccaagaaaccgagcgaaaagaaacgatcgtcgtcgcgtcggcaaccgcagatcgcagcgggcgcgcctcggggctcgccggcgacacccaaggccggcaagtcgcctaaagtctcgcgaccgcctagtgtgccctcg
UL25ms_Seq      175 tcgccctacagcgtctcctcggacgctccgtcgtccttcgagctcgtgcgcgagaccggcggcaccggcgccgccaagaaaccgagcgaaaagaaacgatcgtcgtcgcgtcggcaaccgcagatcgcagcgggcgcgcctcggggctcgccggcgacacccaaggccggcaagtcgcctaaagtctcgcgaccgcctagtgtgccctcg

Towne-UL130r  61000 ctgcccgagaacggcgccggcggcggtggcgacgataacagcagcagcggcggtagcagcagtcgcaccaccagtaacagtagcagaagtaccagtcccgtggcgccaggtgagccgtccgctgccgagggcgatgagtttttccttctgcgacagcgacatcgaagactttgagcgccgaatgctaccgggtcagcgtggccgacaatctg
UL25ms_Seq      385 ctgcccgagaacggcgccggcggcggtggcgacgataacagcagcagcggcggtagcagcagtcgcaccaccagtaacagtagcagaagtaccagtcccgtggcgccaggtgagccgtccgctgccgagggcgtgaagtttttccttctgcgacagcgacatcgaagactttgagcgccgaatgctaccgggtcagcgtggccgacaatctg

Towne-UL130r  61210 ggcttcgagcccagcgtggtcgcgccgcagcacgtcgagtatctcaaattcgtgctgcaagactttgacgtgcagcacctccgccgcctcaacggaatgcatacccatgccggccttcgcgctcaccagcctcgtcgaccccgtcttaaacaacgtagcgcctggcgagcgcgatctcacgcgtcggataatcacgcacgcggtgatcatc
UL25ms_Seq      595 ggcttcgagcccagcgtggtcgcgccgcagcacgtcgagtatctcaaattcgtgctgcaagactttgacgtgcagcacctccgccgcctcaacgtgaacatacccctgaccggccttcgcgctcaccagcctcgtcgaccccgtcttaaacaacgtagcgcctggcgagcgcgatctcacgcgtcggataatcacgcacgcggtgatcatc

Towne-UL130r  61420 aactattactacgtggcgcaaaagaaagcgcgccacgatggtggaggccatacggaccaccgtgcggggcgacacggtacgccaggtagccgcgcaggtcaacaaccagagccgttcggggcgtgcggccgcgctagcgcttcactttctcacgtcacgaaaaggagtgacggacggccagtacgccacgtctctgcggcggctggacgaa
UL25ms_Seq      805 aactattactacgtggcgcaaaagaaagcgcgccacgatggtggaggccatacggaccaccgtgcggggcgacacggtacgccaggtagccgcgcaggtcaacaaccagagccgttcggggcgtgcggccgcgctagcgcttcactttctcacgtcacgaaaaggagtgacggacggccagtacgccacgtctctgcggcggctggacgaa

Towne-UL130r  61630 gagctgcggcatcgcggcacgcccgaatcgccgcggctcaccgaggtctaccagacgctacgcgattacaacgtgctcttctatacccgcccactacacctcgcgcggcgcgctctacctctatcgacaaaacctgcagcggctcaacgagaaccaccgggggcatgctccggctgctttcggtcgaagagatatgcgaagagcacacgctc
UL25ms_Seq     1015 gagctgcggcatcgcggcacgcccgaatcgccgcggctcaccgaggtctaccagacgctacgcgattacaacgtgctcttctatacccgcccactacacctcgcgcggcgcgctctacctctatcgacaaaacctgcagcggctcaacgagaaccaccgggggctgactccggctgctttcggtcgaagagatttgacgaagagcacacgctc

Towne-UL130r  61840 aacgatctggcgttcctagtaggcgtcgagcttatgatcacgcactttcaacgcaccattcgcgtgctgcgctgctatctccagcaccagctgcagagcatctcggagctgtgttacctcatctatgtacaactgccgtcgctgcgcgaagactacgcgcagctcagtgacgtgctctactgggccgtcagtcaaaactacgactacgcg
UL25ms_Seq     1225 aacgatctggcgttcctagtaggcgtcgagcttttgaatcacgcactttcaacgcaccattcgcgtgctgcgctgctatctccagcaccagctgcagagcatctcggagctgtgttacctcatctttgatacaactgccgtcgctgcgcgaagactacgcgcagctcagtgacgtgctctactgggccgtcagtcaaaactacgactacgcg

Towne-UL130r  62050 ctctacgcgagcacgccggcgttgtttgacttttttacgcgtcgtgcgtcagcaggacgccttcatttgcaccgactacgtgtattgcgccctgcgcctgctggcctgtcccgaccgacctattatcggtgacaccggcggcagcagtagctcccaacgcctcgtaggtgagtttatggtgcgcgatccgctgttgcgcgacccgcgcgcc
UL25ms_Seq     1435 ctctacgcgagcacgccggcgttgtttgacttttttacgcgtcgtgcgtcagcaggacgccttcatttgcaccgactacgtgtattgcgccctgcgcctgctggcctgtcccgaccgacctattatcggtgacaccggcggcagcagtagctcccaacgcctcgtaggtgagtttttgagtgcgcgatccgctgttgcgcgacccgcgcgcc

Towne-UL130r  62260 acccacctgcgccagaaactcatcacccgagacatatggtggcgcggttgcaagcgcagccctcgagtcgacacattccggtcgaacacacgggtgtctcctccgtcaccctgctcaaaatctttagccaggtccccccgacgaacgcgaagaagacacgttacgcgagatggctcttaaagcgtttatggaagcgaacggtaatcac
UL25ms_Seq     1645 acccacctgcgccagaaactcatcacccgagacattgacggtggcgcggttgcaagcgcagccctcgagtcgacacattccggtcgaacacacgggtgtctcctccgtcaccctgctcaaaatctttagccaggtccccccgacgaacgcgaagaagacacgttacgcgagtgagctcttaaagcgtttgagaagcgaacggtaatcac

Towne-UL130r  62470 cccgaacaaatctgccgatccccaccaccccgctgccgccgcgcgactatcctcaacgcgacgagcgggaccgtcaccgtcgcgaccgccgcgacagcggggaatactgttgctgatggtggaacgaagcagcagggcggaacggttta
UL25ms_Seq     1855 cccgaacaaatctgccgatccccaccaccccgctgccgccgcgcgactatcctcaacgcgacgagcgggaccgtcaccgtcgcgaccgccgcgacagcggggaatactgttgctga--------------------------------
```

(b)

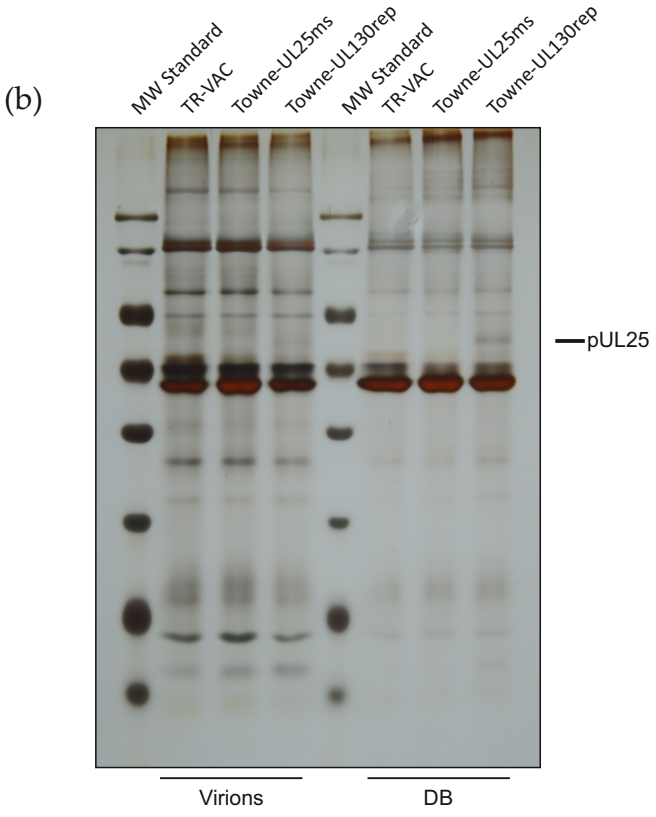

TR-VAC Figure S2

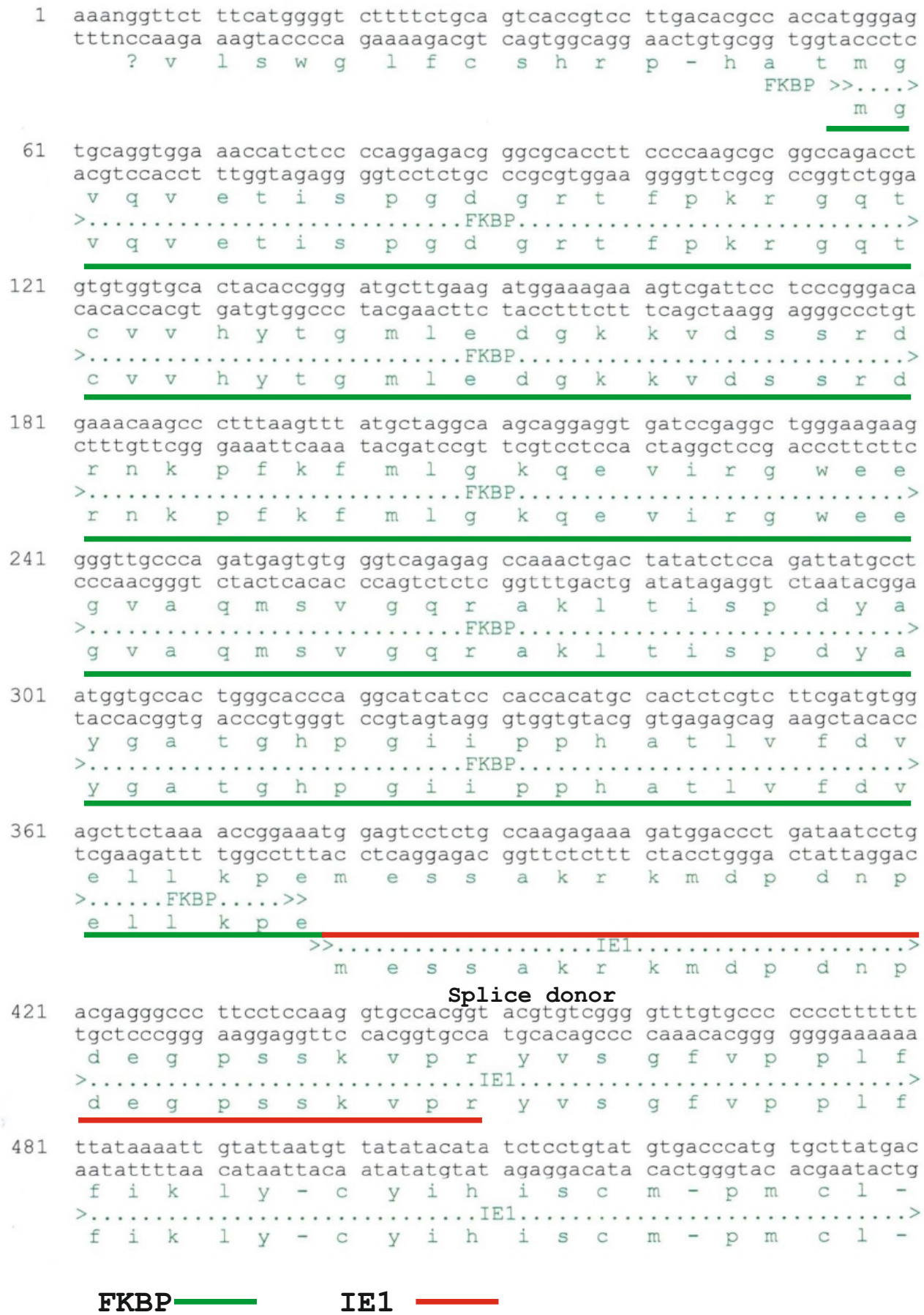

Supplement: Supplementary file 1 [file vaccines-10-01326-s001.zip › vaccines-1863989-supplementary.pdf]
